# Extra Supplement—The Nursing Mirror

**Published:** 1889-01-05

**Authors:** 


### The Nursing Mirror.

All Contributions for this Supplement should be addressed "The Nursing Mirror," care of The Editor, 38, Old Jewry, E.C.

### En Passant.

BRISTOL NURSES' HOME.—The new nurses' home of the British Royal Infirmary has proved a great success already. It not only adds to the comfort of the nurses but has improved their health, and therefore their efficiency. Under these circumstances it is not strange to hear that the committee contemplate further extension, in the shape of a new wing to the home, to hold 25 more nurses' bedrooms. The cost would be about £2,000, but if the wing were built, an institution for supplying private nurses might be started, which would soon prove a source of income to the infirmary.

CHRISTMAS FESTIVITIES.—The nurses of the Nottingham General Hospital had their annual dance on December 18th, a custom which might well be imitated in many other nursing homes. There is no reason why nurses should not have more recreation than they get at present—it would make them better workers. At Nottingham, for instance, Nurse Wood, R.A.M., played piano solos during the scene-shifting of the dramatic entertainment, and on another occasion Nurse Turner sang songs. We don't suppose the matron found either of them worse nurses for their kind efforts. In fact, we rather expect Miss Rimmington urged them on, for she herself is unfailingly energetic and cheerful. In answer to her last appeal she has procured funds to supply the ward with six Matlock invalid couches and two smaller ones. The Christmas Tree on January 2nd was also a grand success, and the whole hospital has enjoyed in very truth a Merry Christmas.

PITY THE POOR PRO.—The question of bullying by nurses is coming to the fore again in the columns of the *Daily News*. Though the wholesale grumbling of the nurses who have written to that paper is childish and absurd, still this question of the treatment of young probationers by older nurses is one which deserves consideration. Last spring one of the minor medical papers started a discussion on the subject, and from letters then printed it was evident that probationers are often severely treated. The very essence of the bullying spirit seems to lie at the bottom of this treatment, for the reason given for unnecessary severity is usually, "I had to undergo it myself, so I do the same by my pros." This is not a golden rule of conduct; this is not a Christian spirit; and the sooner sisters and charge nurses cure themselves of it the better. But on the other hand the helplessness and nervousness of a new probationer is often enough to try the patience of Job; and the sister who always keeps her temper and never speaks sharply, must surely exist in novels alone. It can only be asked of the sister not to give herself over to the theory of bullying being proper treatment for probationers, but to try the theory of curing shyness and ignorance by kindness and teaching. The "Ex-pro." who wrote to the *Daily News* formulates her complaint as follows: "But the 'bullying' was the most trying part, and it was not by any means confined to the staff-nurses, who are for the most part women of small education and scant refinement, but some of the sisters indulge in it as well. It seems to be taken for granted by hospital authorities that new probationers require a thorough 'taking down' in order to fit them for the responsible duties of their calling, that they know nothing, and can do nothing. The unfortunate 'pro.' has to submit to being constantly found fault with; her trifling mistakes are magnified into grave errors, and she is blamed for not knowing small nursing details which, probably, she has never had the chance of learning."

WAGES AND WASHING.—The Whitby Guardians advertised last week for a nurse for their Infirmary, wages £14, without uniform. This announcement did not appear in our list of vacancies; we were not going to insult our readers by placing it there; but we wish to give publicity to this noble offer all the same, and to congratulate the enlightened Guardians of Whitby on their appreciation of the worth of a woman trained in skilled attendance on the sick and dying! Perhaps when some of these same guardians are ill, life hanging in the balance, they will consider a fit attendant to watch and guard them will be amply rewarded by less than a shilling a day; or is it possible that, under the circumstances, these guardians will desire a nurse at three guineas a-week?

A DISORGANISED INFIRMARY.—A nice state of things has been brought to light at the Croydon Union Infirmary, and we may well wonder what will next come come to the fore with regard to the nursing profession. Not long ago a scandal arose through a mistake in burying the body of T. Whiting as that of T. Sennett; of course, such a mistake showed a total lack of management somewhere, and a committee were appointed to investigate affairs. Then it was found that patients were retained as helpers when cured instead of being discharged, nurses and other officials were in the habit of scaling the walls to obtain admittance when late, and the only person found free from blame was the matron. All this is a disgrace to the nineteenth century. There are two ladies on the Croydon Board of Guardians, Miss Shanks and Miss Ely. Where can their eyes have been?

BEDFORD WANTS NURSES.—An extraordinary general meeting of the Governors of Bedford Provident Dispensary has been held to consider the question of starting a Nursing Institute. The attendance was very large, and Mr. Whitbread, M.P., occupied the chair. The adoption of the following report was proposed by Mr. F. Howard, and, after some discussion, was carried: "1. As regards management, we recommend that the Committee of the Bedford Provident Dispensary undertake the carrying on of the Bedford Nurse Institute at the Dispensary premises, for the poor and for private cases. 2. That there be a separate fund for this purpose. 3. That the Dispensary funds be not, in any way, liable for any charges incurred on behalf of the Nurses' Institute. 4. That the Committee of the Bedford Provident Dispensary, whose ordinary meetings are held once a quarter, should appoint a sub-committee of seven of their number, of whom three shall be non-medical men and four shall be medical men on the staff, and that these, together with the secretary, shall form the 'Council of the Nurses' Institute' to carry out the work. 5. Also that a Ladies' Association be formed of all ladies who subscribe 5s. a-year or upwards to maintain the general interests of the institution, and to assist in raising the necessary funds, and three of these, viz., a treasurer and two secretaries, shall be nominated as a visiting committee to take charge of the funds, to assist the lady superintendent in the selection of nurses, confer with her as to domestic arrangements, and help her in carrying on the correspondence. We beg to express our opinion that this work is much needed, and may be very opportunely undertaken at this time, and from the numerous assurances of support and sympathy that we have been given, we confidently believe that the necessary funds will be forthcoming." Mrs. Rawson, present superintendent of the dispensary, was appointed matron of the proposed new branch of work.

### Is a Register for Nurses Desirable?

(By a Medical Practitioner.)

Among modern social developments, hardly any has been more remarkable than that of nursing, which has advanced by leaps and bounds until the craft has grown into a large and important body. Medical men recognise the fact that skilled nursing is often of more value than all the drugs at their command, while there are few cases in which it is not a great aid to successful treatment. The word "nurse," as applied to those who tend the sick, is a term of wide significance. It includes on the one hand the highly-educated specialist, who has had years of medical and surgical work in the wards of a hospital; while on the other hand, it equally describes the "handy woman" of the "Sairey Gamp" type, immortalised by Dickens. An official certificate being an outward and visible sign of competency, at once marks off the qualified class, and on that ground would be a distinct advantage to everyone concerned: to nurses, because the holders could assume their proper position; to the public and to the doctors, because they would be able to single out forthwith the trained women from the mere pretenders. If one may be allowed to use a well-worn simile, registration is like the hall-mark on silver. The quality of the metal is not altered thereby, for, with or without the stamp, silver is always silver. But suppose a metal cream-jug to be placed in the hands of an average member of the British public, ten to one his first act will be to turn that useful object upside down and look for the mark. Some eccentric people may choose to have their silver without any stamp, and there will always be a great many whose pockets will lead them to use electro-plate or britannia metal, both very useful substitutes in their way. Applying the simile, a good nurse remains a good nurse, whether with or without a certificate, but nevertheless, the outward sign will be a guide to those who want the real thing and have not the time, or, it may be, the necessary knowledge, to investigate for themselves. And further, certificated nursing, like stamped silver, should command the highest price in the market. At the same time, the fact that there are always plenty of people who cannot afford the dearer metal should be of some comfort to such as do not possess, and, from the lack of the needful training, are never likely to obtain the certificate.

So far, the chief objection to the scheme seems to be that a register would afford no proof of moral qualities, or of natural gifts, and that any nurse once admitted cannot be struck off its rolls except on the ground of gross misconduct. That is, indeed, an exact representation of the case. Registration has to do with special knowledge and with that only; to certify that the candidate has gone through so many years of training, and passed a test examination of what she has learnt. The certificate does not vouch for general conduct, and is not affected thereby, except in the case of offences of the most flagrant kind. It is the same with law and medicine. Registration takes no notice of the private lives of members of those professions so long as they steer clear of the criminal courts and of infamous conduct. Special knowledge is what the nurses will have to place on record, and not, as some people seem to imagine, such qualities as trustworthiness, self-control, and discretion. Mr. Bonham-Carter asks, "How are these intangible things to be registered?" a question easily answered by pointing out that it would be hardly possible for a probationer to reach any higher rank in her training school, if she failed in the qualities alluded to. Or take the case of a medical man, who, no less than a nurse, requires many virtues outside his special knowledge—are these included in his registered diploma? Certainly not. There is, nevertheless, a powerful safeguard in the consideration that if a doctor fail in morality he is not likely to be successful in practice, any more than the nurse who is wanting in patience and trustworthiness. While the register puts its stamp on professional knowledge, as already stated, it does not vouch for moral qualities. The remedy, however, lies in a nutshell. Let anyone who wants to find out about a nurse write for a character, in confidence if need be, to her former employers.

Before the stage of registration can be reached, the fitness of the candidate must be tested by examinations, and the objection has been raised that such a process would be likely to exclude people who, although not much gifted in the matter of brains, are yet otherwise well fitted for the duties of nursing. It may be at once admitted that a high standard would keep out many excellent recruits, whereas a clever woman could read up enough in a few weeks to "floor" her examiners in a brilliant fashion. The difficulty is a real one, and the way to meet it seems to be to make the tests as practical as possible, to insist on broad principles, and to fix certain limits beyond which questions shall not travel. As competitors increase and examiners get more learned, there is always a tendency to go more and more into detail, hence the modern system of cramming, whereby clever coaches "cram" their pupils with huge masses of undigested facts. Such a course would be fatal to the real spirit of nursing work, of which the handicraft far outweighs the book-learning.

As to the first admission of candidates upon any general register, the conditions would no doubt have to be pretty wide and liberal. Most writers on this subject have instanced the dentists, who, when they got their charter and became a legally-recognised body, had to admit all people regularly practising dentistry at the time. Certainly if every woman actually engaged in nursing were admitted to the register, it would open the door to very serious evils, and it has been well remarked that, on the strength of such loose registration unqualified nurses might go on for twenty or thirty years giving themselves out as duly trained. And although the claims advanced to justify admission call for careful consideration, yet Government would hardly entertain any scheme unless based on the broadest and most liberal terms.

Another important question that is sure to crop up sooner or later is that of general education before entering on hospital work. It is evident that a nurse, at any rate one seeking to pass examinations, must at least know how to read and write. But it would be far from easy to settle how much geography and history, and things of that sort might fairly be asked for at an entrance examination. A woman may make a good poultice without being able to tell one the boundaries of the Mediterranean, or she will dress a burn to perfection, although her mind be an entire blank as to the rule of three and compound interest.

To get a reliable register seems to be a wise and sensible step on the part of a large body of people having common interests to advance and to defend, but it is by no means clear as to how that object may best be attained. A good deal has been said in some quarters about a Royal Charter, regarding the exact nature of which the general idea seems to be rather hazy, so that a word or two on the subject may be added with advantage. In order to obtain the formal recognition of a body with legal powers and privileges one of two methods must be adopted. The first is by Act of Parliament, a roundabout and costly process; and the second is the more direct one, by which the reigning sovereign grants a charter conferring certain rights. Before, however, either king or Parliament can be approached with any prospect of success several conditions have to be fulfilled. The petitioning body must be large, powerful, and united; their demands universally and clearly defined; and it must be shown that the concession would be of distinct advantage to the public at large. Now, with the fullest sympathy for nurses and their welfare, and in all friendliness to the principle of the movement, one may question if it will ever attain to the dignity of a Royal Charter. Some modified scheme will no doubt gradually come into force, springing in the first place from the individual action of the schools themselves, and it is to that quarter one must look for future developments. Let each training school furnish its nurses with a certificate or diploma after they have served their apprenticeship to it and passed a creditable examination—moral, physical, and intellectual. For this privilege let there be a small fee—say five or ten shillings—or better still, let it be a free gift, the name being kept on the roll without payment. A number of official registers will thus be established, and nurses may await with confidence whatever the future may bring forth. In a matter of this sort, to take rash and precipitate action is to invite disaster.

It sounds somewhat trite to say that to establish and organise a corporate body is no light task, yet a glance at some of the subjects involved in any scheme of registration will show their difficulty and importance. Take for example the following :—

A Board of Arbitration elected by all the Nurse Training Schools.

Standard and nature of examinations.

Preliminary education in outside matters.

Expulsion of improper people from the register.

Admission of all existing nurses.

Prosecution of unqualified persons who may wrongfully assume the title.

On the whole, one would be inclined to look upon this movement not so much as putting a stop to bad and unqualified nursing, as a means of stamping the good. The public and members of the medical profession will, no doubt, welcome the day when the nurse who has been properly trained will be able to announce that fact to all and sundry by writing after her name the letters of a title won by honest work, and which will confer on its owner a position at once honourable as it is beyond dispute.

### Lectures to Nurses.

THE Worcester General Infirmary is setting seriously to work to train probationers, and with this good object in view has started two courses of lectures, of which we now give the syllabus, hoping it may prove useful to others. The lectures are illustrated by cases in the wards, and all the instruments and appliances described are shown. Besides the practical ward work, the matron (Miss McLelland) instructs the probationers in ward duties. The introductory lecture was given on November 21st by William Strange, M.D., senior hon. physician. The lectures on "Medical Nursing" are given once a week, by Horace Swete, M.D., hon. physician, in the following order :—

1.—The Organs of Digestion. Anatomy and Physiology. 2.—Assimilation. Principles of Diet. Stimulants. 3.—The Heart and Blood Vessels. Respiration. Air and Ventilation. Drowning and Suffocation. 4.—Excreta. Liver and Kidney. Urine. What to Observe. 5.—The Skin. Baths. The Nurse's duties with regard to it. 6.—The Brain and Nervous System. Paralysis. The Galvanic Battery and its uses. 7.—Medical Emergencies. Poisoning. 8.—Temperature. The Clinical Thermometer. Charting. 9.—Medicines and their administration. 10.—The General Principles of Sanitation. 11.—Infection and Disinfection. Fevers. 12.—Female Diseases. Mental Affections. The Dying.

The lectures on "Surgical Nursing" are given on Monday's by Mr. Rory Fletcher, House Surgeon, in the following order :—

I.—Elementary Anatomy in its application to Nursing. The essentials of a Good Surgical Nurse. Nature the best Surgical Nurse, and some of the methods she adopts. The Bones : Their Arrangements and Uses. The Bones of Children, Adults, and Old People. Rickets. Diseases of Bone and Nursing of "Bone Cases." II.—Fractures : Their Varieties, Nursing, and Treatment. Splints, Varieties, Padding, and Application. The Undressing of Fracture Cases. Fracture Bed and Bed-making. The Prevention of Bed-sores. Bandages : Materials : Varieties and Application, Triangular Bandages, Starch, Plaster, &c., Compound Fractures. After-treatment. III.—Joints : Varieties and Uses. Sprains. Joint Disease. Nursing in "Joint Cases." Hip Disease. Operations on Joints and After-treatment. Strapping of Joints and Extension Apparatus. Operations in general : the room, preparation of patients, instruments, sponges, etc. IV.—Muscles and Tendons : Their Nature and Functions. Paralysis and its Effects. Irritated Muscles. Convulsions. Rigors. Lockjaw. Clubfoot, etc. Ganglia. V.—Connective Tissue and Bursae. Housemaid's Knee, &c. The Circulation : Its Mechanism. The Action of the Heart. Blood-poisoning. Lympathic system. VI.—The Arteries, The Veins. The Capillaries. Bleeding : its Varieties, Causes, Symptoms. and Treatment. The arrest of Hæmorrhage. VII.—Wounds : Their Varieties and Symptoms. Inflammation. Suppuration. Abscess. Sinus. Fistula. Ulceration. How Wounds Heal. The Treatment and Nursing of Wounds. Antiseptics. Ulcers : Their Varieties and Treatment. VIII.—The Complication of Wounds : Gangrene. Erysipelas. Pyæmia. Hectic Burns and Scalds : Varieties and Symptoms. Treatment and Nursing of "Burn Cases." Bed Sores. IX.—The Head. The Scalp, Skull, and Brain. Injuries and Diseases of the Head. The Nursing of "Head Cases." X.—The Spine : Its Structure. Injuries to Spine. Spinal Curvature : Symptoms and Treatment. The Larynx. Tracheotomy. Cut Throat. XI.—The Thorax. Respiration. Fractured Ribs. Wounds of the Chest. The Breast : Its Diseases. The Nursing of "Breast and Chest Cases." XII.—The Abdomen : Its Contents. Injuries to the Abdomen. The Treatment of "Abdominal Cases." Peritonitis. Ovariotomy. Conclusion.

### PRIVATE NURSING.

"YOUR position in the family is a peculiar one. You, as a rule, are only sent for in times of trouble and anxiety ; the nurse, like the physician, is sought when the friends have done their all in caring for the suffering ; when they have reached their limit then we are called upon. You go, not to the house of cheerfulness and rejoicing, but to one of anxiety, sorrow, and sometimes of mourning. The traits of character that are needed by one who takes such a position are of no common order. It is important that you sympathise with those whose trouble you see, and that they feel that you do. Many people seem to have an opinion that sympathy has no place in the composition of those whose profession it is to care for the sick. It always annoys me to hear—as I do hear--it said, 'Oh ! I could not make a good physician,' or 'I could not be a good nurse, I am too sympathetic, and hate to see people suffer.' As if you did not. You know that sympathy does not grow less with your familiarity with suffering. But it is the difference between the helpless and aimless sympathy which is of no benefit to the sufferer and that which is so directed that it is of some avail to the afflicted."—*Dr. Paine.*

### WOMEN AS FUTURE DRUGGISTS.

"IN some of the learned professions the women of this, the last half of the nineteenth century, are walking side by side with its men. The staid old Quaker City has always been the friend of women's progress. The Philadelphia College of Pharmacy has within the past five years graduated four women—Dr. Susan Hayhurst in 1883, Dr. Grace Lee Babb in 1884, Anna Lord in 1887, and Ella Ameron in the present year. Of these Dr. Hayhurst alone is practising in Philadelphia, as druggist, at the Women's Medical College. There are six women now in attendance at the College of Pharmacy ; besides, two are taking a special course in chemistry. Dr. Hayhurst was the pioneer, and made the path easy for those who succeeded her. She entered the College of Pharmacy, and graduated in a class of about one hundred and fifty, of whom she was the solitary woman. Predictions were made that she would not be able to continue to the end, but would encounter opposition from the other members of the class and little encouragement from the professors. She was treated with every consideration, however, and experienced no difficulty in obtaining her degree."—From the *Philadelphia Press.*

### Everybody's Opinion.

### THE PENSION FUND.

J. R. writes: Provincial matrons must be much obliged to Miss Heanley for her few sensible words in your last issue. There is no earthly reason why matrons should not join the Pension Fund, and their example would give courage and confidence to their nurses. It is a great joy to us in the provinces to hear how rapidly the fund is growing, having at last, it seems, triumphed over all its enemies, and risen to the position of security and power which by right belongs to it. At one time I trembled for my profession, lest we should let slip this glorious prize offered so generously to us; now I glory in the fund, and rejoice.

[If the matrons would communicate with the Honorary Manager, Mr. E. Clifford, 8, King Street, E.C., and state the case of their nurses fully, or if nurses and sisters would call themselves at the office, they would find, as all who have taken this step have readily admitted, that the scheme is so carefully elaborated, as to provide for every requirement and circumstance. No one, we are informed, who has conferred with Mr. Clifford, has found the Fund either beyond their means or unable to meet all their wishes. Those who really desire to judge the scheme on its merits, and to do the best for themselves or their nurses, will therefore take an early opportunity of consulting Mr. Clifford, who is courtesy itself.—Ed. *T.H.*]

### WAGES AND WASHING.

A Scotch Nurse writes:—The facts you have given with regard to wages and washing are truly remarkable, and should rouse all nurses to a sense of the weakness of their present position. How we are to increase our wages though, I cannot see, for there is a great rush for every vacancy advertised, and, of course, the cheapest nurses are preferred. Even those who have certificates from a large London hospital are often out of employment, and we who have trained in the provinces can scarcely hope to get regular work again once we leave our parent school. What will happen when we have a register of all persons engaged in nursing, whose mere names being on the register will give them a certain standing with the ignorant, I do not know. Of course, after a time, when the public begins to find out that all sorts and conditions are on the register, and they would do better to demand the certificate of a decent training school, things will recover. But certainly it seems to me that, poorly-paid though we are now, our wages will perforce be lowered when we have to compete with every woman who has been engaged in nursing but has never been trained. I am glad to say the question of washing does not touch us much in the North; it is nearly always done for us.

### PHILANTHROPIC SWEATING.

A Sister writes: I have read with much amusement the letter in the *Daily News* of to-day signed "Ex-Pro." What motive the correspondent can have in thus making her failure public I cannot conceive, but if her letter will deter some of the useless, helpless women who are failures at home and in other walks of life, but who think themselves suited for the noble profession of nursing, she will deserve the gratitude of the whole nursing world. It is a great misfortune that nursing is now so fashionable that people who cannot otherwise make themselves notorious among their friends and acquaintances think they can become heroines by entering a hospital; nor are cases unknown where doctors recommend a few months' experience as a probationer in a hospital as a remedy for hysteria and other nervous disorders. I do not wish to argue that hospital life is ideal, but those whose hearts are in their work will overlook the inconveniences to which "Ex-Pro." refers, and will press forward to the high ideal we may hope they have set before them. No doubt the drawbacks of which "Ex.-Pro." writes will gradually cease to exist; there are many thoughtful men and women who are striving to lighten the burdens of nurses, whose labours will no doubt bear fruit in time, and I, in company with many others in my profession, will confidently leave my fate in their hands. I feel sure I am echoing the wishes of many other nurses in hoping that it may be long before "Ex-Pro." ever again attempts to be a Probationer.

### Appointments.

South Devon Hospital.—Miss F. M. Acheson has been appointed sister of the male ward of this hospital. Miss Acheson trained at King's, and afterwards went to the Radcliffe Infirmary, Oxford. Lately Miss Acheson has been working as an army sister at the Station Hospital, Portsea; and her departure from there is deeply regretted by many friends. All our good wishes go with Miss Acheson to Plymouth, and we hope she may there receive all the appreciation she deserves.

### Presentations.

The nurses of the Private Nursing Institution, Grosvenor Street, Manchester, have presented Mrs. Nicholas, their Lady Superintendent, with a travelling clock, as a token of their sincere regard and esteem. We are always pleased to hear of these tokens of good feeling between nurses and their chiefs.

The weekly board of the Sheffield General Infirmary have presented the late matron (Miss Spencer) with a cheque for £25, as a recognition of her services for 13 years as matron of the institution.

Two ladies were on New Year's Day at Paris the recipients of Crosses of the Legion of Honour. These are Madame Coralie Cahen, who distinguished herself in the ambulances at Metz during the Franco German War, and Sister Eveline, a nun, who has been attached to the Naval and Military Hospital at L'Orient for the space of thirty-five years. Madame Cahen had already the honour of receiving a Cross from the Empress Augusta of Germany in 1872, when the philanthropic French lady was looking after the interests of her countrymen who were lying in German hospitals or prisons.

Note.—In last week's issue, under Armagh Infirmary, read "Sir Patrick Dunn" for "Sir Patrick Drew," and "matron" for "matrons."

### Vacancies.

The following vacancies are announced:—

**Matrons.**

Darenth Imbecile Asylum; £100.

GatesheadChildren's Hospital; £35.

Ulverston Cottage Hospital.

Bootle Borough Hospital; £50.

Southend Victoria Hospital; £30.

Bootle Borough Hospital; £50.

**Nurses.**

Nursing Institution, 96, 97, 98 ', Wimpole Street, London, W., Mr. Wilson has several vacancies for thoroughly-trained Nurses.

Croydon Hospital (Night); £22.

South Shields Union; £25.

Alcester Union; £25.

Southport Trained Nurses' Home.

Nurses' Home, Beckenham.

Royal Albert Hospital, Devonport, Nursing Institute; £25, several.

Eltham Cottage Hospital.

Blackheath Institute for Trained Nurses; £20, several.

Home for Trained Nurses, Bath; £25, several.

Royal Cornwall Infirmary, Truro; £25.

Christ's Hospital, Hertford; £20.

Home for Epileptics, Maghull; £20.

Victoria Home, Bournemouth; several.

Swansea Hospital; £22.

Wallasey Fever Hospital; £25.

Henstead Union; £16.

Home Hospital, Easbourne.

Nursing Institute, Henrietta Street.

Wrexham Infirmary; £20.

Royal Hospital for Women and Children, Waterloo Bridge Road; several.

Nottingham and Notts Nursing Institution; £25, several.

Royal I.O.W. Infirmary, Ryde; £22.

Nurses Home and Training School, Newcastle-on-Tyne; several.

**District Nurses.**

Kensing District Nursing Association; £35.

Cotswold House, Gloucester; £25.

Newlands, Ryde.

Bicester.

**Midwives.**

Parish of Wrexham; £60.
Newlands, Ryde.

**Probationers.**

National Hospital for the Paralysed and Epileptic.

Wrexham Infirmary.

Royal Hospital for Women and Children, Waterloo Bridge Road.

Wallasey Fever Hospital; £15.

Nurses' Home and Training School, Newcastle-on-Tyne; several.

Lancaster Infirmary; £13.

**Parish Nurse.**

Madeley, Newcastle, Staffordshire.

### Notes and Queries.

To Correspondents.—1. Questions may be written on post-cards. 2. Advertisements in disguise are inadmissible. 3. In answering a query please quote the number. 4. If a private answer is desired, a stamped addressed envelope must be forwarded.

**Queries.**

(96) *Nursing in Australia.*—(*a*) A nurse, wishing to go to Australia, will be glad if anyone can tell her the best means to employ in order to obtain an appointment in a hospital there.—J. B. M. (*b*) Is there any opening in Australia for a nurse who has had eighteen months' training in an infirmary, where (*c*) no certificates are given? She has good testimonials. —*Nurse J.*

(93) *Home at Harrogate.*—Is there a convalescent home at Harrogate, where a lady could be received for a short time at a moderate charge? —*C. K. W.*

**Answer.**

(96) *Nursing in Australia.*—See The Hospital, No. 108, page ix. Write to Lady Samuel, 15, Courtfield Gardens, S.W.

(90) *Somerville Club.*—As an ex-nurse, I would like to tell "Middlesex" that I find the Somerville most useful and pleasant. The variety of subjects at the Tuesday evening lectures I think specially useful to nurses, as giving them a change from the round of hospital interests. The food is good and cheap, and the club being open on Sundays, and daily till 10 p.m., is a boon.

(92) *Home for Boys.*—The principal of St. Luke's Home, 97, Merton Road, Wimbledon, is willing to take the boy of 15 at £20 a-year.

